# Reproduction ecology of an emerging fishery resource, the amphibious mudskipper *Periophthalmus chrysospilos*, in the Mekong Delta

**DOI:** 10.1002/ece3.8507

**Published:** 2022-01-24

**Authors:** Quang Minh Dinh, Ton Huu Duc Nguyen, Tran Thi Huyen Lam, Ngon Trong Truong, Tien Thi Kieu Nguyen, Zeehan Jaafar

**Affiliations:** ^1^ Department of Biology School of Education Can Tho University Can Tho Vietnam; ^2^ Department of Molecular Biotechnology, Biotechnology Research and Development Institute Can Tho University Can Tho Vietnam; ^3^ Institute of High Quality Biotechnology‐Food Technology Cuu Long University Vinh Long Vietnam; ^4^ Department of Biology An Khanh High School Can Tho Vietnam; ^5^ Department of Biological Sciences National University of Singapore Singapore Singapore

**Keywords:** batch fecundity, Gold‐spotted mudskipper, gonadosomatic index, length at first maturity, multispawner, spawning season

## Abstract

Populations of *Periophthalmus chrysospilos*, the Gold‐spotted mudskipper, within the Mekong Delta are facing extirpation risks due to indiscriminate harvesting for the growing aquarium and food‐fish trade. This study provides some of the first information on reproductive ecology—such as spawning type and season, length at first maturity, and batch fecundity—of this species, to be used in their management. The sex ratio of wild populations, based on 1031 individuals (523 males and 508 females) is 1:1. The gonadosomatic index (GSI) values are exhibit a non‐normal distribution and changed with gender, season, and site. A combination of GSIs and the monthly appearance of mature gonads suggest that this species reproduces throughout the year, with peak from July to October. This species exhibits sexual and spatial variation in size at first maturity (*L_m_*) as *L_m_* is 6.2–8.6 cm in males and 6.4–7.3 cm in females. The batch fecundity (*F* = 2614 to 23,465 eggs/female) exhibits non‐normal distribution and varies with site, with the highest values at Dam Doi, Ca Mau (13,336 ± 1,279 SE) and the lowest at Tran De, Soc Trang (6654 ± 851 SE). In addition, batch fecundity is directly proportional to body size due to high determination relationships between batch fecundity and fish size (*r* ^2^ > 0.64 for all cases). Information derived on the reproductive biology of this species can inform its conservation, sustainable exploitation, and ex situ propagation.

## Graphical abstract

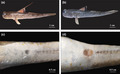

## 1 INTRODUCTION

The gobioid genus *Periophthalmus* (henceforth ‘*Ps*’) comprises 19 species of amphibious fishes, commonly referred to as ‘mudskippers’ (Jaafar & Murdy, 2017). These fishes forage, defend territories, pursue mates, and maintain burrows when the tide is low, on exposed mudflats (Jaafar & Murdy, 2017; Polgar & Crosa, 2009). Popularity of these fishes as pets have soared in the past decades, facilitated by burgeoning global trade in aquaria (Dang & Nguyen, 2009; Monks, 2006; Schäfer, 2005). Traded fishes are not reared in captivity and, therefore, global supply chains are dependent on wild‐caught specimens. In coastal communities in Asia, mudskippers and other gobioid fishes supplement daily protein intake and are popular food fishes (Clayton, 1993; Nguyen, 2000). The extraction rates of these fishes are unknown although many nearshore and estuarine fisheries, where mudskippers naturally occur, are collapsing (Dang & Nguyen, 2009; Udoh et al., 2013). In the Mekong Delta (MD), for example, overexploitation of many fisheries stocks continue unabated (Diep et al., 2014; Trinh & Tran, 2012), and management strategies to recover fish communities are being implemented.

One of the primary elements in fisheries management is to understand the growth and reproductive biology of target taxa (Dinh, Lam, et al., 2021; Fontoura et al., 2009; Miller, 1984; Teichert et al., 2014). The increase in appetite for gobioid fishes and the downward trending fisheries stocks in the MD necessitates a study into the sustainability of these stocks. Yet, knowledge of reproductive biology for gobioid fishes in this region are limited only to a few taxa such as *Parapocryptes serperaster* (Dinh et al., 2016), *Stigmatogobius pleurostigma* (Dinh & Tran, 2018b), and *Butis koilomatodon* (Dinh, Lam, et al., 2021).

The reproductive ecology of mudskippers is complex (see Martin & Ishimatsu, 2017). Unlike other gobioid fishes, courtship displays occur only during the ebb tide, and the mating pair enters a burrow within the mud substrate of the intertidal area, typically excavated by the male (Matoba & Dotsu, 1977). Within the burrow exists an air‐filled nest chamber in which spawning occurs. Eggs are deposited on the ceiling and upper walls of the nest chamber and develop in the air. After the spawning event, females leave the burrow. Males remain with the developing eggs, guarding the burrow and maintaining optimal nest chamber conditions (Martin & Ishimatsu, 2017). Developed eggs hatch when immersed in seawater, the latter introduced into the nest chamber by the attending males (Ishimatsu et al., 2007).

Three *Periophthalmus* species (*Ps*. *chrysospilos*, *Ps*. *Gracilis*, and *Ps*. *variabilis*) occur in Vietnam (Tran et al., 2013). Of these, *Ps*. *chrysospilos* is commonly encountered and have been collected for food and for the aquarium fish trade. This estuarine species naturally occur from the eastern coast of India to Gulf of Thailand, the Java Sea and the MD (Dinh, Nguyen, Lam, et al., 2021; Dinh, Nguyen, Truong, et al., 2021; Jaafar et al., 2009; Kottelat et al., 1993; Le et al., 2021; Murdy, 1989; Murdy & Jaafar, 2017; Tran et al., 2013). The paucity of autecology information on *Ps*. *chrysospilos* presents a challenge when strategizing for sustainable use and conservation of this fishery stock. Our study thus aims to plug this knowledge gap. We report, for the first time, the reproductive ecology of *Ps*. *chrysospilos*, so that informed decisions can be made when utilizing this emerging fishery.

## 2 MATERIALS AND METHODS

### 2.1 Study sites

**FIGURE 1:**
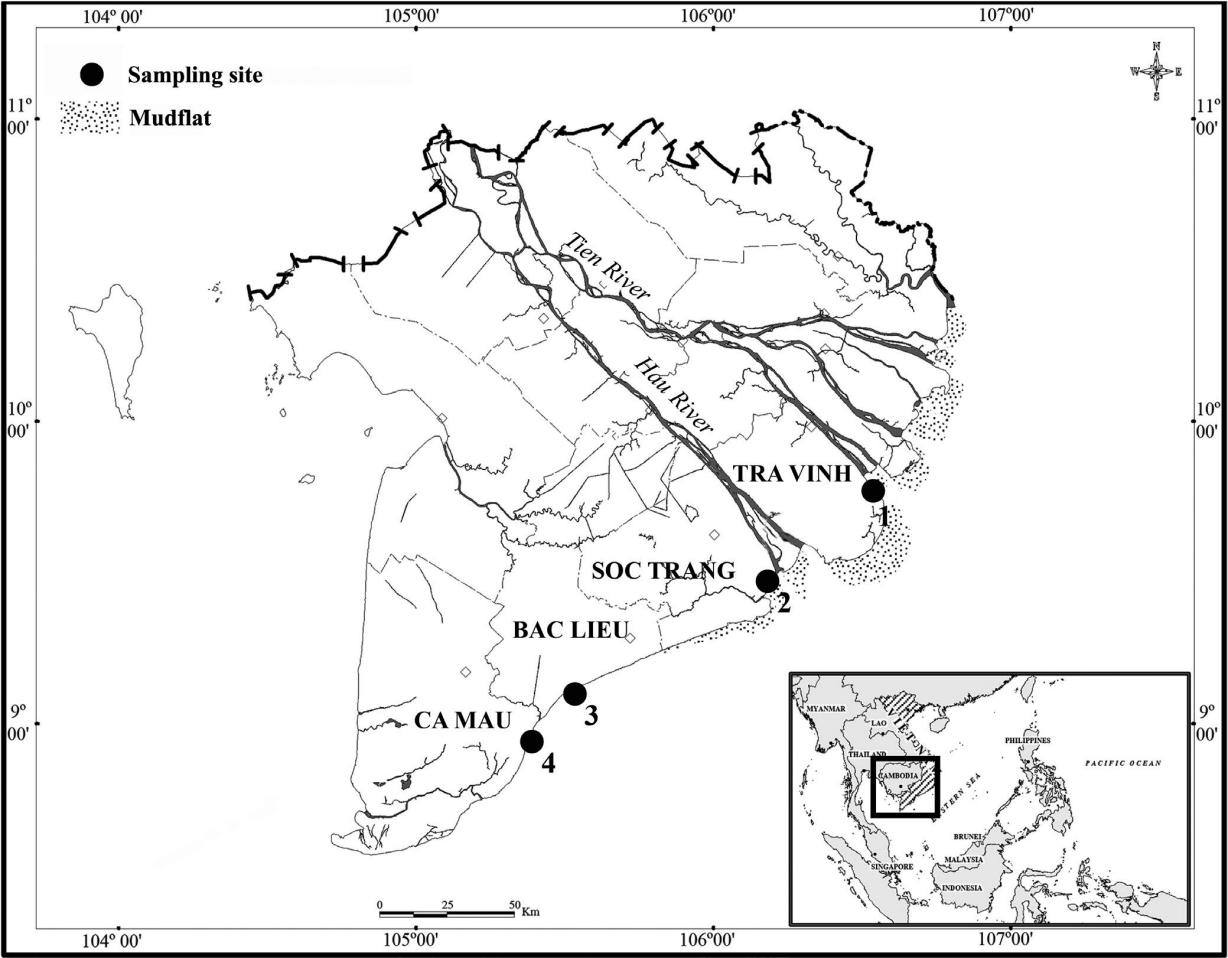
Map of the Mekong Delta showing the sampling sites. (Arrowhead: Sampling area; 1: Duyen Hai, Tra Vinh (9°40'29.5"N 106°34'49.5"E), 2: Tran De, Soc Trang (9°26'19.7"N 105°10'48.1"E), 3: Dong Hai, Bac lieu (9°05'50.5"N 105°29'54.7"E), and 4: Dam Doi, Ca Mau (8°58'10.4"N 105°22'58.9"E) (Dinh, 2018))

This study was carried out from April 2020 to March 2021 at four estuarine areas—Duyen Hai, Tra Vinh (9°40'29.5"N 106°34'49.5"E; TV), Tran De, Soc Trang (9°26'19.7"N 105°10'48.1"E; ST), Dong Hai, Bac Lieu (9°05'50.5"N 105°29'54.7"E; BL), and Dam Doi, Ca Mau (8°58'10.4"N 105°22'58.9"E; CM) (Figure 1). These four sites experience semi‐diurnal tidal cycle and daily mean temperature of approximately 27°C. Precipitation rates are extremely low during the dry season (January–May) accounting for only about 10% of the total annual rainfall, while the wet season (June–December) receives approximately 400 mm of rain per month (Le et al., 2006). The pH ranged from 7.7 to 7.9, varying with site; but pH within site remained constant between seasons. The salinity varied widely from 11.2‰ to 26.2‰, remaining constant within site, but varying significantly with seasons (Dinh, Lam, et al., 2021). The primary tree species at these sites are the mangal taxa *Avicennia marina* and *Sonneratia caseolaris* (Nguyen et al., 2020).

Fishes were caught by hand over the final three days of each month. This species is easily distinguishable from sympatric congeners by the extent of fusion of the pelvic fin and the pattern of the first dorsal fin (Murdy and Jaafar (2017)). Tricaine methanesulfonate (10 g/L) was used to anesthetize the fish specimens which were washed under the tap and preserved in 5% buffered formalin before transport to the laboratory. The use of fish in the present study is assessed and approved by The Council for Science and Education, School of Education, Can Tho University (Animal Welfare Assessment number: BQ2020‐03/KSP).

### 2.2 Examination of fish samples

**FIGURE 2:**
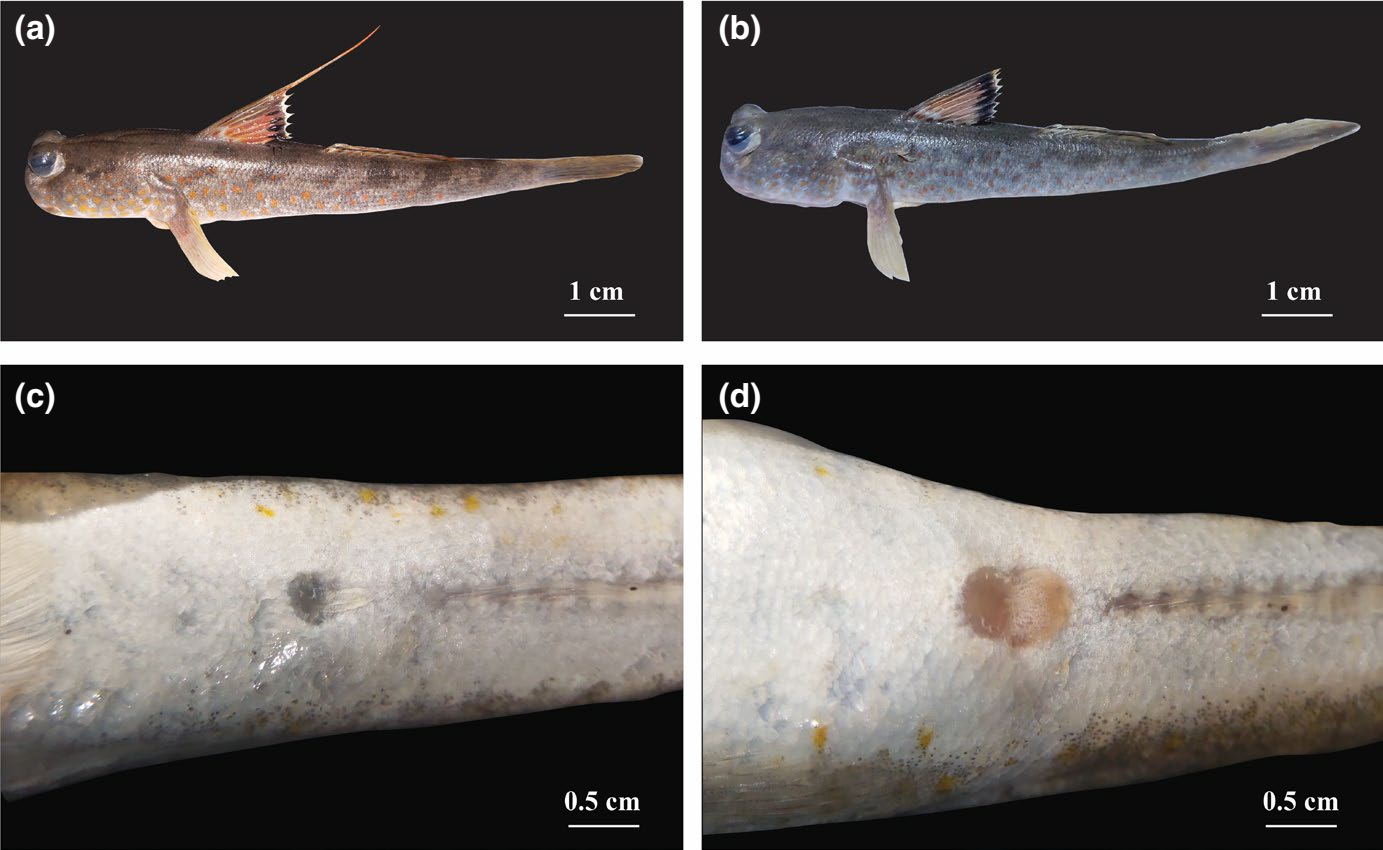
The genital papillae of *Periophthalmus chrysospilos* (a: male individual; b: female individual; c: genital papilla of male; d: genital papilla of female)

In the laboratory, sex of specimens was determined from the urogenital papillae (Figure 2). In males, the papillae are narrow, broader at the base, and taper toward a pointed tip. In females, the papillae are broad, and similar in width at the base and tip. The total length (TL to the nearest 0.1 cm) and weight (W to the nearest 0.01 g) were then measured and recorded. The ovaries and testes were then removed and weighed to the nearest 0.01 mg before classifying these into six developmental stages following the methods used in Dinh, Tran, Ngo, et al. (2020). Twenty‐five ovaries (stages I to V, five samples/stage) and 20 testes (stages I to IV, five sample/stage) were selected and stained according to the method of Carleton et al. (1980). The ovaries (stages I to V, 30 samples/stage) and testes (stages I to IV, 30 samples/stage) at each stage were cut into three subsamples—one at each end and one in the middle. After, the diameters of 150 ovarian subsample and 120 testicular subsamples were measured using Motic Images Pro Plus 2.0 software (Dinh et al., 2016), and oocyte and spermatocyte stages were described following Yamamoto (1956) and Yamazaki (1965).

The equation GSI = 100 × (*G*/*W*) (*G*: gonad weight, *W*: fish body weight; Sturm, 1978) was applied to calculate the gonadosomatic index (GSI). The fish reproductive season was estimated from a combined analysis of GSI and gonad frequency occurrence (Alonso‐Fernández et al., 2011; Dinh & Le, 2017).

Fish length at first maturity (*L_m_*) of males and females at each site was calculated from the formula: *P* = 1/(1 + exp[−*r* × (TL–*L_m_*)]) (*P*: proportion of mature individuals in a length class; TL: fish total length; and *r*: model parameter) (Zar, 1999).

Batch fecundity, the number of oocytes laid per spawning, was estimated based on the gravimetric method (Hunter et al., 1985). In this study, 66 mature ovaries were used to estimate the batch fecundity as *F* = (*n* × *G*)/*g* (*n*: number of oocytes in subsample; *g*: weight of subsample; and *G*: ovarian weight) (Bagenal, 1967). Three tissue subsamples of 1mm thick were extracted from each ovary, two samples from each end and one sample from the midovary. Each slice was weighed to the nearest 0.01 mg, and oocytes were separated with tap water and a spear needle in a Petri dish. Thereafter, all mature oocytes were counted under the magnifier.

### 2.3 Data analyses

The Shapiro–Wilk test was used to evaluate the normal distribution of GSI and fecundity (Kim, 2015). If GSI was a normal distribution, a *t*‐test with the Levene test for equality of variances was used to verify the variation of GSI between gender and seasons at each site, whereas the Mann–Whitney test was used otherwise. The equality of variances of GSI among 4 sites and 12 months was confirmed by the Levene test that was also used to verify the equality of variances of batch fecundity (*F*) between four sites. One‐way ANOVA with Tukey's post hoc was used to test the change in GSI for sites and months when the GSI variances were equal, but one‐way ANOVA with Tamhane's T2 was analyzed if the GSI variances were not. In contrast, Kruskal–Wallis test was performed to verify if GSI varied with site and months when GSI variable did not display normal distribution. This test was also applied to confirm the variation of *F* among four sites if *F* was not a normal distribution. The logarithmic regression was applied when testing relationships of fish size (TL and *W*) and *F* (Metin et al., 2011). SPSS software v.21 was used for data analyses, and all tests were set at a 5% significance level.

## 3 RESULTS

### 3.1 Sex ratio

A total of 1,031 individuals (523 males and 508 females) were sampled over 1 year at four sampling sites (Table 1). From April 2020 to March 2021, the largest number of fish samples were collected from BL (303 individuals), while the fewest was from CM (229 individuals, Table 2). The samples of males and females were approximately equal (*χ^2^*, *p* > .05 for all cases, Table 2) both in the wet and dry seasons (see Table 2) at TV; ST and BL. However, at CM, the ratio of males to females was about 1.5:1.

**TABLE 1:** Number of *Periophthalmus chrysospilos* individuals sampled at each study site for each month

Sampling year	Sampling month	Duyen Hai–Tra Vinh		Tran De–Soc Trang		Dong Hai–Bac Lieu		Dam Doi–Ca Mau	
		Male	Female	Male	Female	Male	Female	Male	Female
2020	April	20	8	6	14	9	21	7	8
2020	May	10	7	12	12	12	8	11	6
2020	June	7	17	5	28	17	17	13	12
2020	July	14	7	19	9	12	18	16	15
2020	August	7	14	5	5	17	11	15	6
2020	September	13	6	13	4	26	16	11	9
2020	October	3	14	11	9	7	9	6	5
2020	November	6	11	13	7	14	5	11	6
2020	December	7	12	8	15	8	16	10	6
2021	January	9	9	10	16	5	15	11	6
2021	February	11	6	9	12	12	8	11	5
2021	March	13	8	9	9	7	13	15	8
Total		120	119	120	140	146	157	137	92

**TABLE 2:** Distribution of male and female specimens of *Periophthalmus chrysospilos* at each study site

Sampling sites	Duyen Hai, Tra Vinh	Tran De, Soc Trang	Dong Hai, Bac Lieu	Dam Doi, Ca Mau	Dry season	Wet season	Total
Male	120	120	146	137	209	314	508
Female	119	140	157	92	199	309	523
Sex ratio	1.01:1.00	0.86:1.00	0.93:1.00	1.49:1.00	1.05:1.00	1.02:1.00	0.97:1.00
χ^2^	0.01	1.54	0.40	8.84	0.25	0.04	0.22
*p*	.95	.22	.53	.01	.62	.84	.64

### 3.2 Spermatogenesis

**FIGURE 3:**
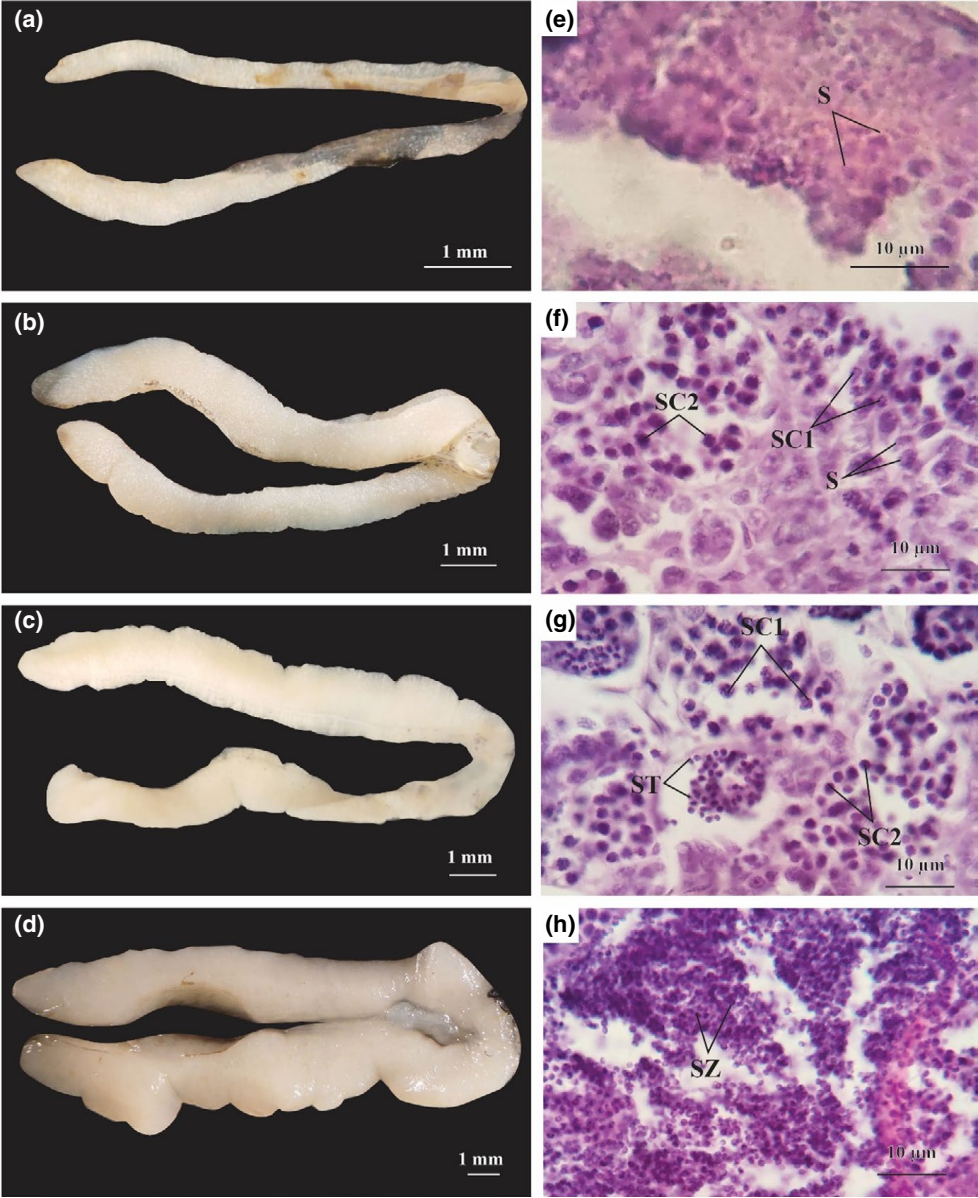
Testicular morphology and histology of *Periophthalmus chrysospilos* (a, b, c, and d: external morphology stages I–IV of testis; e, f, g, and h: histology stages I–IV of testis; S, Spermatogonia; SC1, primary spermatocytes; SC2, secondary spermatocytes; ST, spermatid; SZ, spermatozoa)

In stage I, the testes were elongate, smooth, and measured 0.42 ± 0.01 mm in diameter. These were milky in appearance and easily confused with stage I ovaries (Figure 3a). At this stage, the testes contained mainly spermatogonia (S) which were basophilic with the dark purple of hematoxylin (Figure 3e). Testes increased to 0.88 ± 0.01 mm diameter in stage II (growing stage), and appeared milky white, long, and slender (Figure 3b). At this stage, the testes comprised mainly primary spermatocytes (SC1), secondary spermatocytes (SC2), and a few spermatogonia; nuclei of the SC1 stages were duskier than those of SC2 (Figure 3f). At stage III (maturing stage), testes were 1.19 ± 0.02 mm in diameter, smooth, elongate, light yellow, and with obvious sperm ducts (Figure 3c). At this stage, the testes comprised mainly spermatids (ST) and a few SC1 and SC2 in their lobules (Figure 3g). Testes at stage IV (mature stage) were smooth but swollen with prominent blood vessels, and measured 2.39 ± 0.04 mm in diameter (Figure 3d). Testicular lobules were enlarged and filled with sperms. A group of spermatozoa (SZ), which are tiny cells with globe‐shaped nucleus stained with hematoxylin, was produced in the testicular cavities and sperm ducts. Spermatids and a few SC2 were also found in testes at this stage. Spermatozoa were released by mature males in this period (Figure 3h). This study did not recover male individuals exhibiting stages V (degenerating stage) and VI (recovery stage).

### 3.3 Oogenesis

**FIGURE 4:**
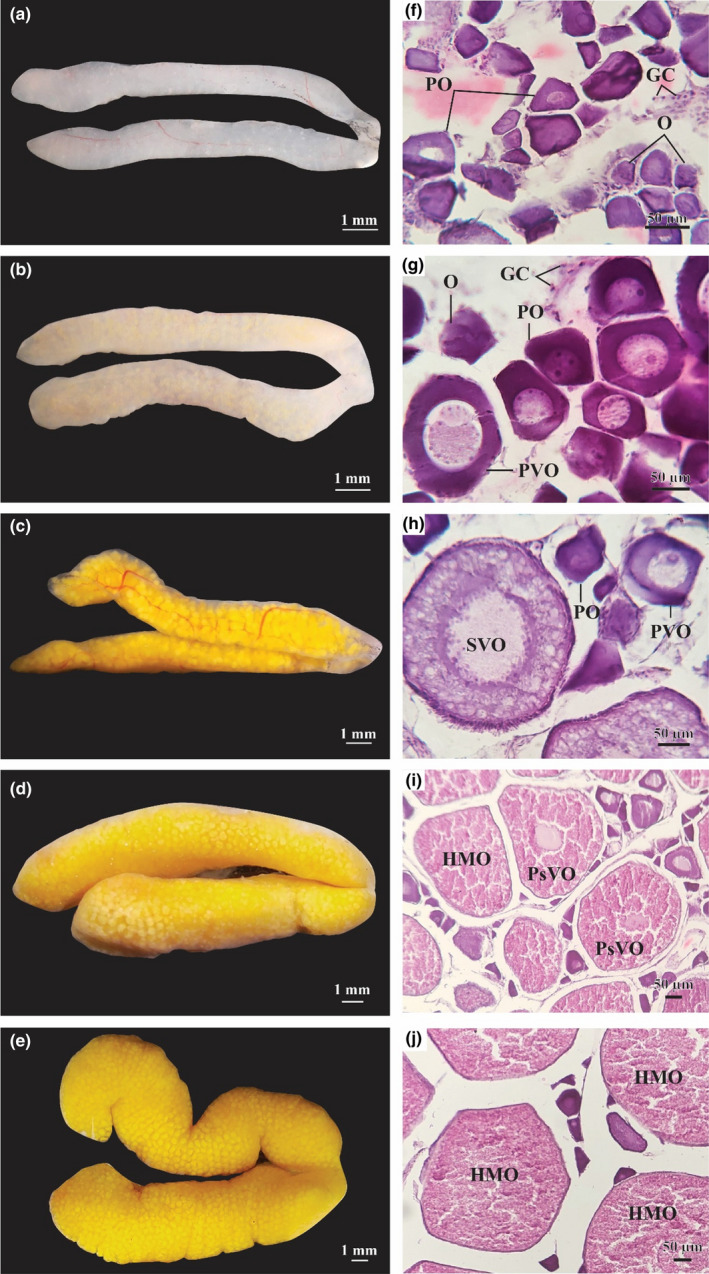
Ovarian morphology and histology of *Periophthalmus chrysospilos* (a, b, c, d, and e: external morphology stages I–V of ovary; f, g, h, I, and k: histology stages I–V of ovary; GC, germ cells; O, oogonia; PO, primary oocyte; PVO, primary vitellogenic oocytes; SVO, secondary vitellogenic oocytes, PsVO: post vitellogenic oocytes, HMO: hydrated oocytes)

Ovaries in stage I (early growing stage) were thin, smooth, pale white, and measured 1.16 ± 0.02 mm in diameter (Figure 4a). At this stage, the ovaries mostly contained germ cells (GC), oogonia (O), and a few primary oocytes (PO) (Figure 4f). At stage II (growing stage), the ovaries became pale yellow and increased to 1.46 ± 0.03 mm in diameter (Figure 4b). At this stage, O, PO, and primary vitellogenic oocytes (PVO) with several yolk granules in the cytoplasm were found in the ovaries; all Os were small, dark purple, with prominent cytoplasm while POs were larger with bright nucleus (Figure 4g). At stage III (maturing ovary), the ovaries were light yellow, smooth, with protruding blood vessels, and measured 1.87 ± 0.04 mm in diameter (Figure 4c). The ovaries in this stage contained O, PO, PVO, and secondary vitellogenic oocytes (SVO) while the nuclei and yolk were accumulated. Some oocytes were in the oil droplet stage and contents of cytoplasm were visible. The yolk granules and oil droplets accumulated in the cytoplasm stained more eosin‐basophilic (Figure 4h). In stage IV (mature stage), ovaries were long, smooth, and measured 2.70 ± 0.03 mm in diameter (Figure 4d). Individual yellow eggs can already be observed. The ovaries consisted mainly of post vitellogenic oocytes (PsVO) and some hydrated oocytes (HMO) as well as some PO, PVO, and SVO (Figure 4i). In stage V (ripe stage), the swollen ovaries were surrounded with prominent blood vessels, reaching a diameter of 4.04 ± 0.02 mm. The circular yellow eggs were visible to the naked eye and separated easily from each other (Figure 4e). The ovaries in this stage consisted of primarily post vitellogenic oocytes (PsVO) and hydrated oocytes (HMO) as well as some PO, PVO, and SVO (Figure 4f). The oil droplets and yolk granules were partially homogeneous while the nuclei contracted, and the nuclear membranes faded away. The nucleoli were near the center of the nuclei; the former could be observed effortlessly in PsVO, but not in HMO. In the present study, ovaries in stage VI (recovery stage) were not found in the female samples.

### 3.4 Seasonality and gonadosomatic indices

**FIGURE 5:**
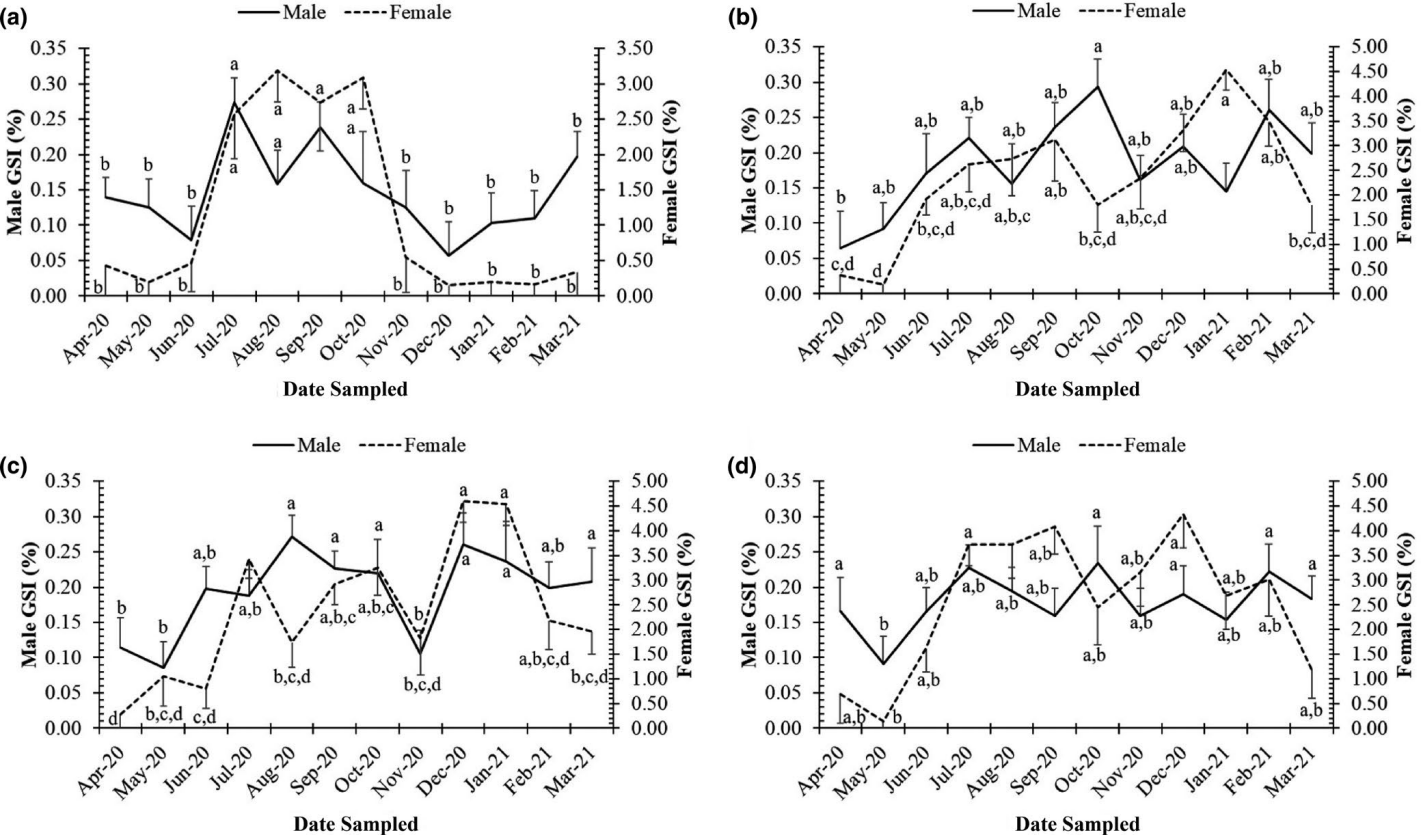
Gonadosomatic indices of males and females of *Periophthalmus chrysospilos* at study sites. (a, b, c, and d represent Duyen Hai, Tra Vinh; Tran De, Soc Trang; Dong Hai, Bac Lieu; and Dam Doi, Ca Mau)

The GSI values were found to exhibit a non‐normal distribution (Shapiro–Wilk test, NJ = 0.87, *p* < .01) and changed by gender (Mann–Whitney U, *Z* = 27.04), season (*Z* = 6.17, *p* < .01) and site (Kruskal–Wallis H, *χ^2^* = 40.72, *p* < .01). Higher GSI values were recorded in the wet season compared to the dry season for both sexes at all four sites (Figure 5).

At each site, the GSI values of females were significantly higher than those of males (Mann–Whitney U, *p* < .01 in all cases) and GSI values of males and females showed monthly fluctuations (Kruskal–Wallis H, *χ^2^*_male_ = 147.19, *χ^2^*_female_ = 29.74, *p* < .01). Notably, mean values of GSI in females were the highest in August 2020 at TV (3.19 ± 0.45 SE); in January 2021 at ST (4.53 ± 0.41 SE); in December 2020 at BL (4.60 ± 0.43 SE); and in September 2020 at CM (4.08 ± 0.56 SE). The higher GSI values (1.71 ± 0.18 SE at TV, 2.41 ± 0.20 SE at ST, 2.75 ± 0.23 SE at BL and 3.24 ± 0.35 SE at CM) in females were observed during the wet season. Similar patterns were observed in males, where GSI values were highest in the wet season from July to October. For example, the highest mean of male GSI was found in July 2020 at TV (0.27 ± 0.03 SE); in October 2020 at ST (0.29 ± 0.04 SE); in August 2020 at BL (0.27 ± 0.03 SE); and in October 2020 at CM (0.23 ± 0.05 SE).

**FIGURE 6:**
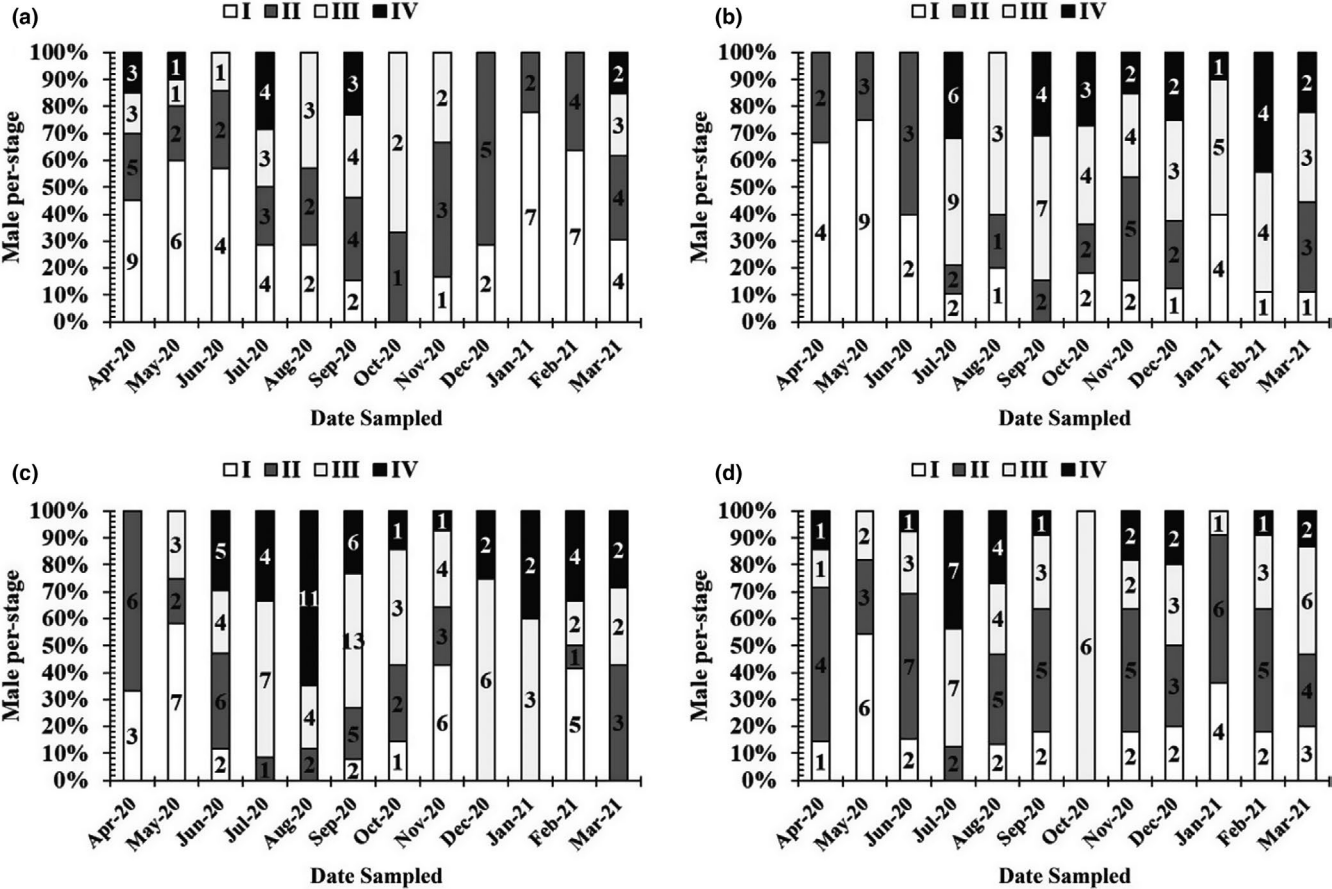
Gonadal stage composition of male *Periophthalmus chrysospilos*. (The number in a column represents fishes at each gonad development stage; a, b, c, and d represent Duyen Hai, Tra Vinh; Tran De, Soc Trang; Dong Hai, Bac Lieu; and Dam Doi, Ca Mau)

**FIGURE 7:**
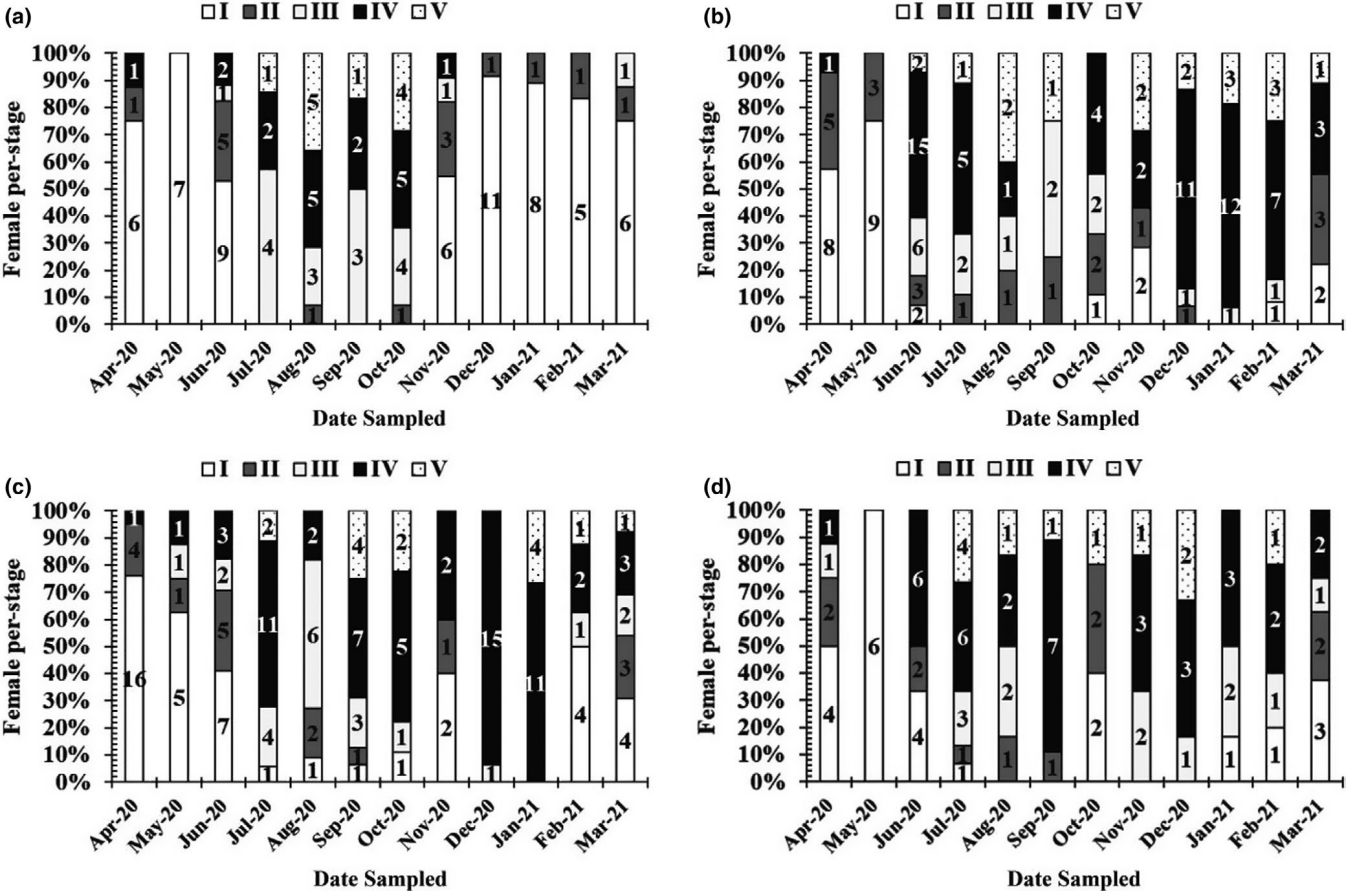
Gonadal stage composition of female *Periophthalmus chrysospilos*. (The number in a column represents fishes at each gonad development stage; a, b, c, and d represent Duyen Hai, Tra Vinh; Tran De, Soc Trang; Dong Hai, Bac Lieu; and Dam Doi, Ca Mau)

Mature males and females, with gonads in stage IV, were found almost monthly (Figures 6 and 7), thus indicating that this species reproduces throughout the year, with a peak during the wet season.

### 3.5 Length at first maturity and fecundity

**FIGURE 8:**
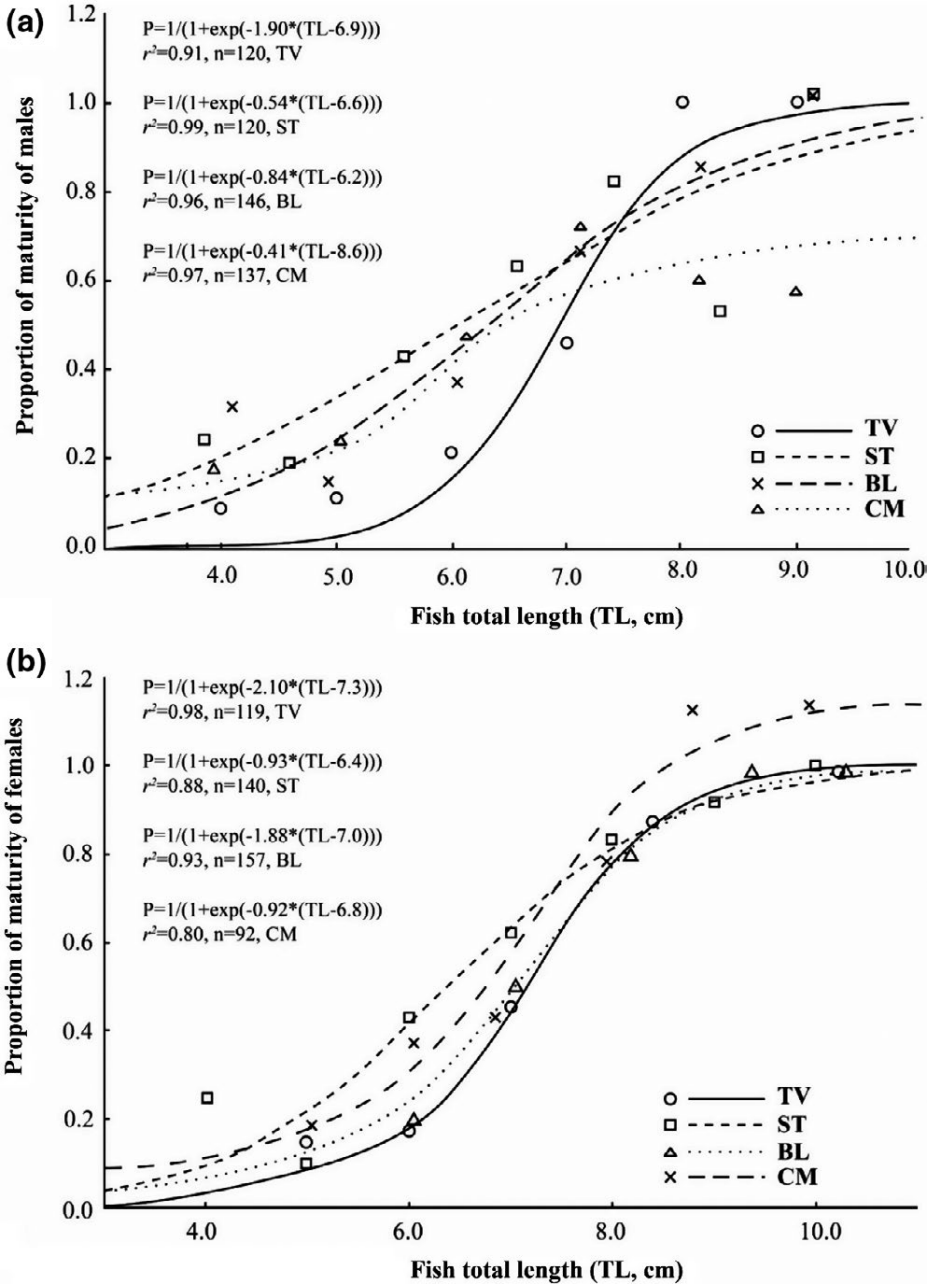
Size at first maturity of male (a) and female (b) *Periophthalmus chrysospilos*. (TV, ST, BL, and CM represent Duyen Hai, Tra Vinh; Tran De, Soc Trang; Dong Hai, Bac Lieu; and Dam Doi, Ca Mau, respectively)

**FIGURE 9:**
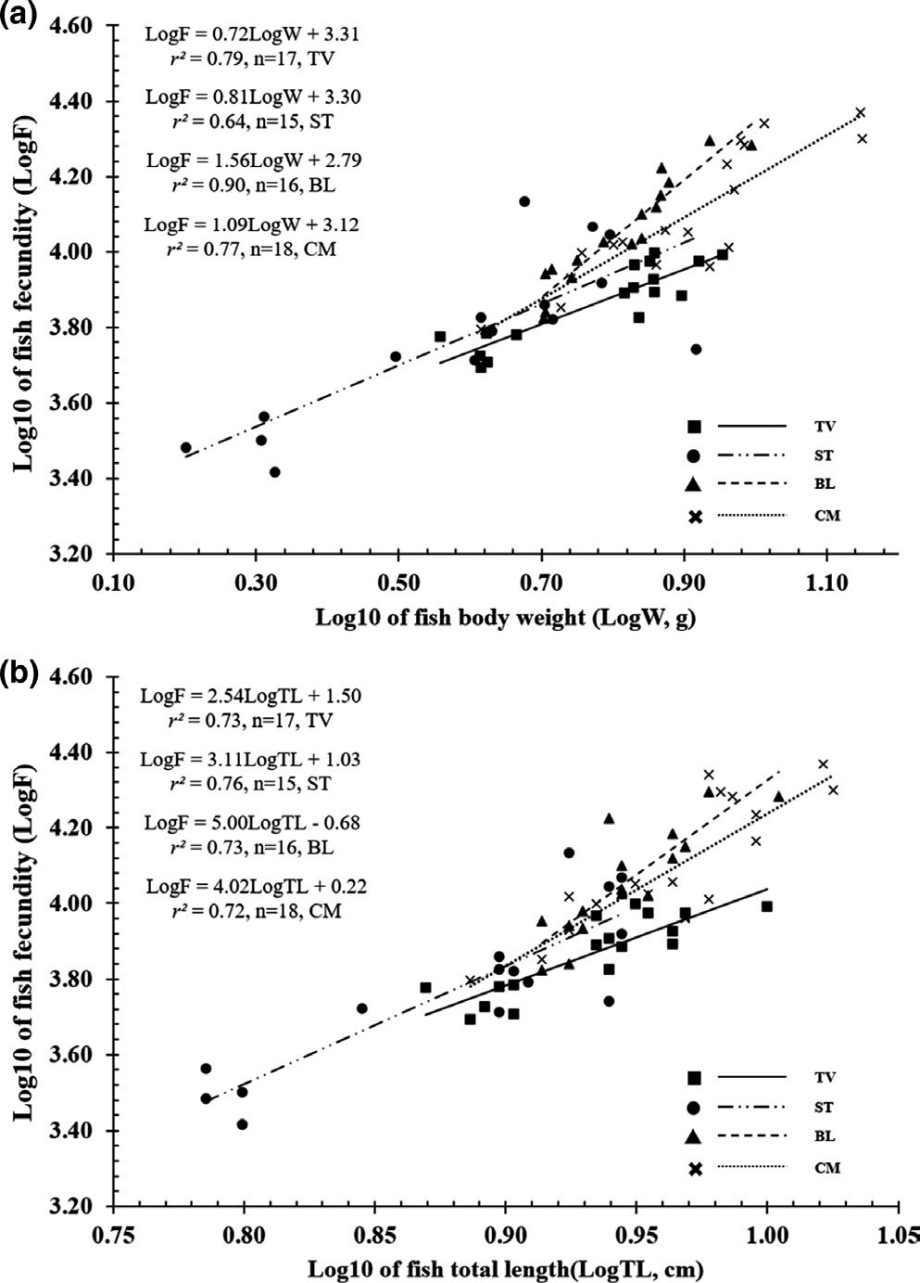
Relationships between fecundity and fish weight (a) and total length (b) of *Periophthalmus chrysospilos* (TV, ST, BL, and CM represent Duyen Hai, Tra Vinh; Tran De, Soc Trang; Dong Hai, Bac Lieu; and Dam Doi, Ca Mau, respectively)

The length at first maturity (*L_m_*) of *Ps*. *chrysospilos* differed between the four sites; this value fluctuated for both male and female individuals from 6.2 to 8.6 cm and 6.4 to 7.3 cm, respectively (Figure 8). The *F* exhibited non‐normal distribution (Shapiro‐Wilk Test, NJ = 0.96, *p* < .01) and varied between sites, fluctuating between values 2614 eggs/female and 23,465 eggs/female (Kruskal–Wallis H, *χ^2^* = 35.55, *p* < .01). The highest average fecundity values were from individuals recovered from BL (12,012 ± 1015 SE) and CM (13,336 ± 1279 SE), while the lowest values were from those caught from TV (7516 ± 418 SE) and ST (6654 ± 851 SE). The *F* values are positively correlated with total length and weight due to the high *r^2^* value of the relationships between *F* and fish size (>0.64 for all cases; Figure 9).

## 4 DISCUSSION

This study extensively assessed the reproductive biology of *Ps*. *chrysospilos* from the MD. Examination of gonads recovered from specimens collected over 12 months revealed the presence of mature males and females, with gonads at stage IV, for every month from every site. This indicates that *Ps*. *chrysospilos* reproduces throughout the year. However, reproduction is hypothesized to peak in the wet season, from July to October, based on the high values of GSI in both males and females during this time. Mekong Delta, where the fishes were caught, is an alluvial plain with abundant food sources and spawning grounds (Nedeco, 1993). Within the MD, other mudskippers such as *Boleophthalmus* (henceforth *B*.) *boddarti* (Dinh et al., 2015) and *Periophthalmodon* (henceforth *Pn*.) *septemradiatus* (Dinh, Tran, Ngo, et al., 2020) also show increased rates of reproduction during the wet season. In tropical areas, peak reproduction in many fish species occurs in the wet season, likely due to increased accumulation of nutrients resulting from the high rainfall (Blaber, 2000; Elliott et al., 2007; Whitfield, 1990). As with *Ps*. *chrysospilos*, peaks in reproduction is also observed during the wet season for other mudskippers such as *Ps*. *barbarus* from the Imo estuary, Nigeria (Etim et al., 2002), and *Pn*. *schlosseri* in Malaysia (Mazlan & Rohaya, 2008).

The appearance of spermatids (ST), secondary spermatocytes (SC2), and spermatozoa (SZ) in stage IV of mature testes; as well as oogonia (O), primary oocytes (PO), primary vitellogenic oocytes (PVO), and secondary vitellogenic oocytes (SVO) in stage IV of mature ovaries indicate that *Ps*. *chrysospilos* is multiple spawner; a reproductive strategy observed in many gobioid species (Miller, 1984). This mode of reproduction is also exhibited in other mudskipper species such as *B*. *boddarti* (Dinh et al., 2015), *Pn septemradiatus* (Dinh, Tran, Ngo, et al., 2020), and *Ps barbarous* (Etim et al., 2010). Mudskippers have also been shown to be serial spawners—eggs are laid multiple times within one breeding period, for example, *Ps barbarus* in Nigeria (Chukwu et al., 2010).

Environmental parameters affect the batch fecundity of many fish species. The *F* of *Ps*. *chrysospilos* (2614–23,465) changed significantly between the four sites in this study—the highest fecundity was recovered at CM (*F* = 6248–23,465) while the lowest was at TV (*F* = 4927–9941). In gobioid fishes, *F* values can also vary greatly from one species to another. For instance, one of the lowest *F* was reported from *Eviota lacrimae* with only 100 eggs, while one of the highest was reported from *Awaous guamensis* with approximately 500,000 eggs (Ha & Kinzie, 1996). Intraspecies variation in *F* values is high; for example, *F* values in individuals of *B*. *boddarti* were between 2100 and 12,300 in India but between 9800 and 33,800 in Vietnam. Batch fecundity values positively correlate to body length and weight (Song & Baek, 2005). In our study, *F* of *Ps*. *chrysospilos* was positively correlated with fish body size; however, this correlation was only moderate (*r* ^2^ > 0.64 in all cases). Gobioid species sympatric with the gold‐spotted mudskipper within the MD also had a positive correlation between fertility and fish body sizes such as *Butis butis* (Dinh & Le, 2017), *Trypauchen vagina* (Dinh, 2018), *Stigmatogobius pleurostigma* (Dinh & Tran, 2018a), and *Pn*. *septemradiatus* (Dinh, Tran, Tran, et al., 2020). The sex ratio of *Ps chrysospilos* was not biased in favor of females for the survey period from April 2020 to March 2021. Equal sex ratio was also reported in other mudskipper species such as *Pn*. *schlosseri* in Malaysia (Mazlan & Rohaya, 2008), *Ps barbarus* in Nigeria (Etim et al., 2010), and *B*. *boddarti* (Dinh et al., 2015) in the MD. In contrast, the sex ratio between males and females of 1.5:1.0 was recovered at only one site —in CM—for this study. Unequal sex ratios have been reported in five *Pn*. *septemradiatus* populations in the MD, in which the ratio of males to females was reported to be 1.4:1.0 (Dinh, Tran, Ngo, et al., 2020). The factors contributing to the unequal sex ratio was not accounted for in this or past studies in the MD, but males are hypothesized to be more than females since they compete for mates, build shelters, and guard eggs (Fraser et al., 2001; Gutowsky & Fox, 2011).

Males and female mudskippers mature at different lengths (Dinh et al., 2015; Dinh, Tran, Ngo, et al., 2020). Moreover, in species that guard eggs or young, the length at first maturity (*L_m_*) tends to be longer. The length of maturity for *Ps*. *chrysospilos* is similar to *Ps*. *barbarus* (Etim et al., 2010) and *Pn*. *septemradiatus* (Dinh, Tran, Ngo, et al., 2020), but slightly shorter than *B*. *boddarti* (Chandran et al., 2014; Dinh et al., 2015). This trend is also observed in *Pomatoschistus marmoratus*, in which the *L_m_* in males, who are key egg guarders, is longer than in females (Mazzoldi et al., 2002). In *Ps*. *chrysospilos*, in which the males are also egg guarders, the length at first maturity of males is longer than females; for example, in CM, *L_m_* of males is 8.6 cm while *L_m_* of females is 6.8 cm).

In many parts of the world, mudskippers are exploited for consumption and the aquarium trade. Mudskippers are eaten throughout Indo‐China and Japan by coastal communities. Within the Imo River Estuary in southeast Nigeria, approximately 79% of the population of *Ps*. *barbarus* is harvested for the aquarium trade (Udoh et al., 2013). In Vietnam, mudskippers feature in many local dishes. They are in high demand for food and are increasingly targeted for aquaria, yet the rate of extraction of many species remains unknown. Furthermore, Vietnam reports losses of mangrove areas of up to 42% of original cover (FAO, 2007). This additional impact of mudskipper populations obligate to mangrove habitats is poorly understood. Elucidating the reproductive biology of mudskippers is the first step toward achieving a sustainable extractive management plan as capture can be regulated for size, and for periods outside of peak spawning. The reproductive biology of mudskippers is also of interest to aquarists, and culture fisheries.

In conclusion, this study revealed that *Ps*. *chrysospilos* populations are reproductively viable throughout the year but with a peak reproduction rate during the wet season. Fecundity was found to differ with locality, and significantly correlated with body size. In males, the size at first maturity was longer than that of females, and the *L_m_* values differed with site. This information can effectively contribute toward a holistic and cogent conservation plan for mangrove areas, and organisms, in Vietnam.

## CONFLICT OF INTEREST

None declared.

## AUTHOR CONTRIBUTIONS

**Quang Minh Dinh:** Conceptualization (equal); Data curation (equal); Formal analysis (equal); Funding acquisition (equal); Investigation (equal); Methodology (equal); Project administration (equal); Resources (equal); Software (equal); Supervision (equal); Validation (equal); Visualization (equal); Writing – original draft (equal); Writing – review & editing (equal). **Ton Huu Duc Nguyen:** Data curation (equal); Formal analysis (equal); Investigation (equal); Methodology (equal); Resources (equal); Software (equal); Writing – original draft (equal); Writing – review & editing (equal). **Tran Thi Huyen Lam:** Formal analysis (equal); Investigation (equal); Resources (equal); Writing – original draft (equal); Writing – review & editing (equal). **Tien Thi Kieu Nguyen:** Conceptualization (equal); Investigation (equal); Writing – original draft (equal). **Ngon Trong Truong:** Investigation (equal); Writing – review & editing (supporting). **Zeehan Jaafar:** Conceptualization (equal); Investigation (equal); Methodology (equal); Validation (equal); Visualization (equal); Writing – original draft (equal); Writing – review & editing (equal).

## Data Availability

Please find our raw data at https://doi.org/10.5061/dryad.2fqz612q8
